# Impaired self-other differentiation in frontotemporal dementia due to the *C9ORF72 *expansion

**DOI:** 10.1186/alzrt145

**Published:** 2012-08-13

**Authors:** Laura E Downey, Colin J Mahoney, Martin N Rossor, Sebastian J Crutch, Jason D Warren

**Affiliations:** 1 Dementia Research Centre, UCL Institute of Neurology, University College London, London, UK

## Abstract

**Introduction:**

An expanded hexanucleotide repeat in the *C9ORF72 *gene has recently been identified as an important cause of frontotemporal dementia and motor neuron disease; however, the phenotypic spectrum of this entity and its pathophysiologic basis have yet to be fully defined. Psychiatric features may be early and prominent, although a putative cortico-thalamo-cerebellar network has been implicated in the pathogenesis of the clinical phenotype. Differentiation of self from others is a core cognitive operation that could potentially link network disintegration with neuropsychiatric symptoms in *C9ORF72*-associated frontotemporal dementia.

**Methods:**

We undertook a detailed behavioral analysis of self-other attribution in a 67-year-old male patient with behavioral variant frontotemporal dementia (bvFTD) due to the *C9ORF72 *expansion by using a novel paradigm requiring differentiation of the effects of self- and non-self-generated actions. The patient's performance was assessed in relation to two older male patients with bvFTD not attributable to the *C9ORF72 *expansion and four healthy older male subjects.

**Results:**

Compared with the healthy control group, the patient with the *C9OFR72 *mutation showed a deficit of self-other differentiation that was disproportionate to his otherwise relatively indolent clinical phenotype. The performance of the other patients with bvFTD was similar to that of healthy subjects.

**Conclusion:**

We propose that impaired self-other differentiation is a candidate mechanism for neuropsychiatric decline in association with the *C9ORF72 *expansion. We offer this preliminary observation as a stimulus to further work.

## Introduction

An expanded hexanucleotide repeat in the *C9ORF72 *gene has recently been identified as an important cause of frontotemporal dementia and motor neuron disease [1-3]; however, the phenotypic spectrum of this entity and its pathophysiologic basis have yet to be fully defined. Psychiatric manifestations including delusions, hallucinations, and severe anxiety disorders have been identified as frequent and prominent and may be presenting features [1,3,4]. Although detailed neuroanatomic-phenotypic correlation has yet to be undertaken in the *C9ORF72 *mutation spectrum, a culprit cortico-thalamo-cerebellar network has been identified as a potential substrate for certain clinical features, in particular, for neuropsychiatric symptoms [1]. Differentiation of self from others is a core cognitive operation that could potentially link network disintegration with neuropsychiatric symptoms in *C9ORF72*-associated frontotemporal dementia. Disordered self-other differentiation has been implicated in the pathogenesis of various psychiatric conditions, including schizophrenia, out-of-body autoscopic experiences, anxiety, and depression [5-8]. Neuroimaging evidence in the healthy brain suggests that the cerebellum is a key component of distributed cortico-subcortical circuitry that represents and calibrates the effects of actions generated by oneself and others [5,9].

We recently had the opportunity to investigate this issue in a patient, NT, with the *C9ORF72 *expansion, who exhibited an indolent phenotype of behavioral variant frontotemporal dementia (bvFTD) with relative preservation of many cognitive abilities. We designed a novel behavioral paradigm specifically to assess NT's ability to distinguish between the effects of self- and non-self-generated actions. We assessed NT in relation to a group of healthy older male control subjects and two older male patients (DC1 and DC2) with bvFTD not attributable to the *c9ORF72 *expansion.

## Methods

### Case descriptions

#### NT

NT is a 67-year-old right-handed male retired information technology consultant with a postgraduate degree qualification. He presented with an insidious decline in personality and behavior extending over some 20 years. His increasing impassivity, impulsivity, and distractibility had led to his retirement from work. His wife reported that he was less gregarious and less empathic, more obsessional about money, and showed an increased preference for sweet foods. He made tactless remarks to strangers and had only limited insight into his difficulties. No concern had been expressed about his episodic memory or route-finding abilities, and his language, though somewhat tangential, remained articulate. No history suggested delusions, hallucinations, or other perceptual disturbance. A family history existed of behavioral decline in NT's mother from age 50, and dementia with parkinsonism in a maternal uncle. NT's general neurologic examination was unremarkable; in particular, no features of motor neuron disease were found. Longitudinal neuropsychological assessment over a 9-year interval demonstrated a largely stable profile with superior performance in most cognitive domains and only a mild relative weakness of aspects of executive function; NT's general neuropsychological data at the time of the experimental assessment are summarized in Table 1. NT fulfilled current consensus criteria for a syndromic diagnosis of bvFTD [10].

**Table 1:** Demographic and neuropsychological characteristics of patients and healthy controls

	NT	DC1	DC2	**Healthy controls**^a^ (*n *= 4)
Age (years)	67	63	74	67 (6.03)
Education (years)	15	12	16	15 (2)
				
General intellect				
WASI VIQ	118	63	115	72 (3.4)
WASI PIQ	132	91	111	57 (2.6)
NART (/50)	>75th	<25th	>90th	41 (4.8)
				
Episodic memory				
RMT Words (/50)	25-50th	<5th	5-10th	44 (5.2)
RMT Faces (/50)	>95th	<5th	<5th	46 (6.1)
Executive function				
D-KEFS Stroop word	>50th	>50th	>50th	21 (3.5)
D-KEFS Stroop Inhibition	>50th	>50th	>50th	53 (15.5)
				
Semantic processing				
BPVS (/150)	146	103	138	149 (1.5)
				
Other skills				
GNT (/30)	>50th	<1st	<1st	27 (2.9)
Forward DS (/12)	>95th	75th-90th	90th-95th	8 (1)
Reverse DS (/12)	>50th	50th-75th	90th-95th	6 (1)
Addition	>95th	>50th	25-50th	7 (1.2)
Subtraction	>95th	25th-50th	>50th	8 (3.3)
VOSP	>95th	25-50th	>50th	19 (0.5)
CBI	52	203	46	n/a
Experimental conditions^b^				
Self (/10)	10	10	10	9.5 (9-10)
Synchronous (/10)	3	7	5	6.5 (5-7)
Asynchronous (/10)	4	9	10	9.5 (9-10)

Percentile ranking scores of neuropsychological performance are shown. Raw scores are listed where percentile scores are not available or applicable. BPVS, British Picture Vocabulary Scale; CBI, Cambridge Behavioural Inventory total score (across domains including mood, beliefs, challenging behaviors, disinhibition, eating, sleep, stereotypies, motivation, and insight (Wedderbum *et al.*, 2008)); D-KEFS Stroop (word and inhibition), Delis-Kaplan Executive Function System; DS, digit span; GNT, Graded Naming Test; n/a, not applicable; NART, National Adult Reading Test; RMT, Recognition Memory Test; Verbal Intelligence score; VOSP, Visual Object and Space Perception battery; WASI PIQ, Wechsler Abbreviated Scale of Intelligence-Performance Intelligence score; WASI VIQ, Wechsler Abbreviated Scale of Intelligence, ^a^Mean (standard deviation) values shown, except for experimental conditions, in which mean (range) values shown. ^b^See text for explanation.

**Figure 1:**
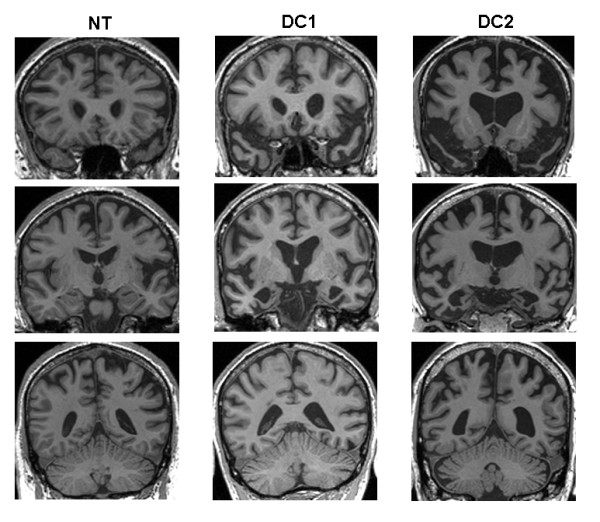
**Representative coronal T_1_-weighted MR brain sections for the patient with *C9ORF72*-associated frontotemporal dementia (NT) and for patients with non-*C9ORF72*-associated frontotemporal dementia (DC1 and DC2)**. Sections have been selected to capture the anterior frontal lobes and temporal poles (top row), anterior perisylvian regions and medial temporal lobes (middle row), and posterior parietal lobes and cerebellum (bottom row). Scans were acquired at the time of the behavioral assessment. The left hemisphere is shown on the right in all sections.

Serial registered *T*_1_-weighted volumetric brain MRI demonstrated progressive, diffuse cerebral and cerebellar volume loss; brain MRI at the time of the behavioral assessment (Figure 1) showed mild atrophy, predominantly affecting left frontal and perisylvian regions. Peripheral electrophysiological studies revealed no evidence of anterior horn cell dysfunction or peripheral neuropathy. Genomic screening identified a pathologic hexanucleotide expansion in the *C9ORF72 *gene.

#### DC1

DC1 is a 63-year-old right-handed male retired plumber. He presented with a 6-year history of progressive personality change, initially with uncharacteristic aggressive outbursts and supervening social withdrawal, obsessionality, behavioral rituals, and sweet tooth. Memory impairment was another prominent early feature. No history of delusions, hallucinations, or other perceptual disturbance was present. The patient had a known family history of early-onset frontotemporal dementia with autosomal dominant inheritance. His general neurologic examination was normal. In particular, no features of motor neuron disease were noted. Serial neuropsychological assessments over a 6-year interval demonstrated progressive impairment, particularly affecting naming and executive functions; DC1's general neuropsychological data at the time of the experimental assessment are summarized in Table 1. DC1 fulfilled current consensus criteria for a syndromic diagnosis of bvFTD [10].

Serial registered *T*_1_-weighted volumetric brain MRI demonstrated progressive cerebral atrophy, most marked in the anteromesial temporal lobes, which were relatively symmetrically affected (Figure 1). Genomic screening demonstrated a c.1216C>T (p.Arg406Trp) mutation of the microtubule-associated protein tau (*MAPT*) gene.

#### DC2

DC2 is a 76-year-old left-handed male retired professor of English literature. He presented with an 11-year history of progressive prosopagnosia and personality change with social disinhibition and obsessionality. No history of delusions, hallucinations, or other perceptual disturbance was noted, with no known family history of dementia. The general neurologic examination was normal. Serial neuropsychological assessments over a 6-year interval demonstrated a largely stable profile with prominent anomia and recognition memory dysfunction; DC2's general neuropsychological data at the time of the experimental assessment are summarized in Table 1. DC2 fulfilled current consensus criteria for a syndromic diagnosis of bvFTD [10].

Serial registered *T*_1_-weighted volumetric brain MRI demonstrated progressive cerebral volume loss involving the anterior temporal and frontal lobes, more marked on the right (Figure 1). Genomic screening excluded a *C9OR72 *mutation.

### Healthy control subjects

Four healthy right-handed male control subjects (mean age, 67 years; range, 58 to 72 years; see Table 1) matched to NT for age (*t*_5 _= 0.04; *P *> 0.05) and with similar educational and social backgrounds but no history of neurologic or psychiatric illness also were assessed.

This study was approved by the local research ethics committee under Declaration of Helsinki guidelines. All subjects gave informed consent to participate, and provided consent to publish.

### Experimental assessment

**Figure 2:**
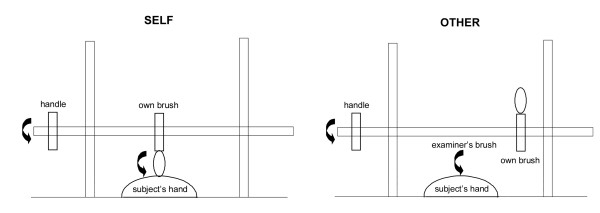
**Schematic diagram of the experimental setup in the "self" and "other" action task conditions**. See text for further explanation.

The experimental set-up is schematized in Figure 2. A paintbrush (14.5 × 1 cm, 1"25 was suspended by using a cross-clamp from a rod positioned between two table-mounted retort stands, such that the rod (and the attached paintbrush) could be rotated freely by manipulating a handle attached to one end. The subject was positioned with his dominant hand resting palm-down on the table between the retort stands, and the apparatus was adjusted so that the paintbrush lightly tracked across the skin of the hand when the handle was rotated by the subject, using his nondominant hand. During the experiment, the paintbrush was randomly moved along the suspending rod from trial to trial, such that the brush either would contact the subject's hand ("self" condition) or would not contact the subject's hand ("non-self" trials); on "other" trials, the experimenter delivered the tactile stimulus by using an identical paintbrush, either in time with the subject's own action (synchronous condition) or with a short delay (around 1 second; asynchronous condition). The retort-mounted paintbrush was shifted by the experimenter before every trial (whether self or non-self) to minimize any extraneous cues from sound or the absolute position of the brush. The subject was blindfolded and instructed to rotate the handle 3 times in every trial: the task on each trial was to decide whether the brush stimulus was generated by the subject's own action or by that of the experimenter. It was established before commencing the experiment that subjects were able reliably to detect the sensory stimulus delivered by the brush. Thirty experimental trials were administered, comprising 10 self, 10 non-self synchronous, and 10 non-self asynchronous trials in randomized order. Subject responses were recorded and stored for offline analysis. No time limit was imposed, and no feedback about performance was given during the test.

Patients' performance was compared with that of healthy control subjects by using the Crawford and Howell modified one-tailed *t *test for significant differences in single-case studies [11] and with a significance threshold of *P *< 0.05.

## Results

Results on the experimental behavioral test are summarized in Table 1. Compared with healthy controls, NT exhibited a significant deficit of self-other differentiation over all experimental conditions (*t_5 _*= -3.48; *P *< 0.021). Analyses of the three experimental conditions separately revealed that NT's performance was flawless for attribution of self-generated actions, whereas he performed significantly worse than healthy controls for attribution of both synchronous, non-self-generated actions (*t_5 _*= -4.05; *P *< 0.008) and asynchronous, non-self-generated actions (*t_5 _*= -5.5, *P *< 0.001). NT's errors were therefore entirely overattributions of the experimenter's actions on non-self trials as self-generated responses. Like NT, patients DC1 and DC2 correctly attributed all self-generated actions. However, in contrast to NT, DC1 and DC2 showed no significant differences in performance relative to healthy controls on either of the non-self conditions (DC1: overall *t_5 _*= 0.12, synchronous *t_5 _*= 0.45, asynchronous *t_5 _*= -0.78; DC2: overall *t_5 _*= -0.36, synchronous *t_5 _*= -1.34, asynchronous *t_5 _*= 0.78; all *P *> 0.05), and each patient's performance fell within the control range for every condition.

## Discussion

It is evident from the range of scores (Table 1) that healthy controls and patients alike performed generally rather poorly on the synchronous non-self condition; performance differences were exposed largely in the asynchronous condition. This pattern of results suggests that the experimenter was able to control accurately the degree to which the external stimulus simulated (or did not simulate) the effect of a self-generated action.

## Conclusions

Here we have shown that differentiation of the effects of one's own versus others' actions may become impaired in frontotemporal dementia associated with the *C9ORF72 *expansion. Indeed, the deficit of self-other differentiation appeared disproportionate to NT's otherwise relatively mild cognitive phenotype. As this patient's cognitive evolution was otherwise so indolent, we hypothesize that impaired self-other differentiation may be a key feature in the development of the complex behavioral disturbances that accompany the *C9ORF72 *expansion, or may to lead the development of more-typical cognitive deficits. The specificity of this finding for *C9ORF72*-associated frontotemporal dementia remains to be resolved. However, the present data suggest that the ability to distinguish one's own from others' actions is not comparably affected in other forms of frontotemporal dementia (including *MAPT*-associated disease); nor does it appear to be simply a consequence of more severe disease, as DC1 and DC2 were both substantially more cognitively impaired than was NT. Together, these findings raise the possibility that impaired self-other differentiation is a behavioral signal of *C9ORF72 *mutations. It is of interest that NT tended to "overattribute" others' actions to his own agency. A similar overattribution bias to self for actions of ambiguous origin has been reported both in healthy individuals [12,13] and in schizophrenia [14]. Both healthy controls and the two patients without the *C9ORF72 *expansion here made self-overattribution errors in the synchronous non-self condition, but (unlike NT) were able to use the increased temporal delay in the non-self asynchronous condition to identify the external origin of the action. Although the overattribution to self of external actions may seem somewhat paradoxic in a condition such as schizophrenia with delusions of external control, it has been argued [6] that such a deficit might lead to an impaired ability to model one's own versus others' actions and an abnormal sense of invasion by external forces masquerading as oneself.

Previous theoretic accounts of the neurobiologic basis for sense of agency [9,15] have accorded the cerebellum a key role as a comparator of efferent and afferent motor commands. However, the interpretation of agency is likely to engage a distributed brain network also including thalamus and posterior parietal cortex for transmission and integration of the sensory consequences of actions and prefrontal and cingulate cortex for cognitive appraisal of integrated percepts [16]. Impaired ability to distinguish the effects of one's own from others' actions might therefore potentially result from impaired prediction coding in the cerebellum or defective integration of sensory percepts by the thalamus or the parietal or prefrontal cortex [5,7,17]. The elements of this distributed network have been implicated in neuroimaging [1,18] and neuropathologic [4,18,19] studies of patients with *C9ORF72 *expansions. Although neuroanatomic correlation was not possible here, we hypothesize that dysfunction of the previously delineated cortico-thalamo-cerebellar network may have underpinned the behavioral deficit of self-other differentiation exhibited by NT [5,7,9]. Although impaired self-other action attribution has not, to our knowledge, been proposed previously as a general mechanism of behavioral decline in frontotemporal dementia, complex behavioral phenotypes remain poorly characterized in pathophysiologic terms. It may be that a range of more basic deficits is expressed in broadly similar behavioral phenotypes, within which certain features (for example, early prominent neuropsychiatric symptoms) may give a more-specific clue to the key pathophysiologic mechanism at work with particular mutations. We propose that impaired self-other differentiation in patients with *C9ORF72 *mutations may index a generic mechanism of defective own-action modeling and representation that may be somewhat analogous to the deficit proposed previously in patients with schizophrenia (6). Such a pathophysiologic mechanism could potentially be expressed in a range of clinical neuropsychiatric phenomena.

We present this single-case analysis with a number of caveats and suggestions for future work. Foremost among these, the findings require replication in a cohort of patients with the *C9ORF72 *expansion, with both clinical and neuroanatomic correlation. This patient did not exhibit clinically overt psychotic symptoms (delusions, hallucinations, or other perceptual disturbances); it would be particularly pertinent to test our hypothesis in a group of patients who do exhibit such symptoms (for example, delusions). The proposed role of the putative cortico-thalamo-cerebellar network could be directly assessed in a structural or functional brain-imaging paradigm. The use of other tasks designed to explore self-other differentiation in this population would further corroborate the present findings. The specificity of the deficit for *C9ORF72*-associated frontotemporal dementia requires further corroboration through comparison with larger cohorts of patients representing other forms of genetic and sporadic frontotemporal dementia. In addition, within the *C9ORF72 *expansion group, a need exists to establish the time course of development of the deficit in a longitudinal analysis. If our conjecture is correct, impaired self-other differentiation might manifest as an early feature, possibly even presymptomatically. The paradigm we propose is relatively straightforward and could be adapted to group longitudinal applications. We hope that our preliminary observation stimulates further hypothesis-led work directed to establishing the pathophysiologic basis of the complex behavioral disorders that characterize this newly discovered entity.

## Abbreviations

BPVS: British Picture Vocabulary Scale; bvFTD: behavioral variant frontotemporal dementia; CBI: Cambridge Behavioural Inventory; D-KEFS: Delis-Kaplan Executive Function System; DS: digit span; GNT: Graded Naming Test; MRI: magnetic resonance imaging; NART: National Adult Reading Test; PIQ: Performance Intelligence Quotient; RMT: Recognition Memory Test; VIQ: Verbal Intelligence Quotient; VOSP: Visual Object and Space Perception; WASI: Wechsler Abbreviated Scale of Intelligence.

## Competing interests

We have no financial competing interests to declare. LED, CJM, SJC, and JDW receive salary and research support from the Medical Research Council, Alzheimer Research UK, and the Wellcome Trust. MNR sits on the Data Monitoring Committee for Servier DMC Phase 2B AD Study S38093, and also sits on the Bapineuzumab Independent Safety Monitoring Committee for Janssen Al/Pfizer.

## Authors' contributions

All authors read and approved the manuscript. LED was involved in study planning, design, and coordination, acquired and analyzed the behavioral data, and was involved in drafting and critically revising the article. CJM was involved in study design, acquisition of neuroimaging data, and drafting and critically revising the article. MNR assessed the patients clinically and was involved in study design and in drafting the article. SJC and JDW obtained funding for and supervised the study, and were involved in study planning and design, in data collection, and in drafting and critically revising the article.
